# Proprioception Is Necessary for Body Schema Plasticity: Evidence from a Deafferented Patient

**DOI:** 10.3389/fnhum.2016.00272

**Published:** 2016-06-16

**Authors:** Lucilla Cardinali, Claudio Brozzoli, Jacques Luauté, Alice C. Roy, Alessandro Farnè

**Affiliations:** ^1^The Brain and Mind Institute, University of Western Ontario, London, ONCanada; ^2^ImpAct Team, Lyon Neuroscience Research Center, INSERM U1028, CNRS UMR5292, LyonFrance; ^3^University of Lyon, LyonFrance; ^4^Hospices Civils de Lyon, Hôpital Neurologique, Mouvement et Handicap and Neuro-immersion, LyonFrance; ^5^Aging Research Center, Department of Neurobiology, Care Sciences and Society, Karolinska Institutet, StockholmSweden; ^6^Laboratoire Dynamique Du Langage, CNRS UMR 5596, LyonFrance

**Keywords:** tool-use, deafferentation, body schema, kinematic, grasping

## Abstract

The ability of using a large variety of tools is important in our daily life. Behind human tool-use abilities lays the brain capacity to incorporate tools into the body representation for action (Body Schema, BS), thought to rely mainly on proprioceptive information. Here, we tested whether tool incorporation is possible in absence of proprioception by studying a patient with right upper-limb deafferentation. We adopted a paradigm sensitive to changes of the BS and analyzed the kinematics of free-hand movements before and after tool-use, in three sessions over a period of 2 years. In the first session, before tool-use, the kinematics of the deafferented hand was disrupted. Similarly, the first movements with the tool (a mechanical grabber elongating the arm by ~40 cm) showed an abnormal profile that tended to normalize at the end of the session. Subsequent free-hand movements were also normalized. At session 2, 6 months later, the patient exhibited normal free-hand kinematic profiles, additionally showing changes in grasping kinematics after tool-use, but no sign of tool incorporation. A follow-up 2 years later, further confirmed the normalized kinematic profile but the absence of tool incorporation. This first description of tool-use in absence of proprioception shows the fundamental role of proprioception in the update of the BS. These results provide an important further step in understanding human motor control and have implications for future development of rehabilitation programs for patients with sensory deficits.

## Introduction

**FIGURE 1:**
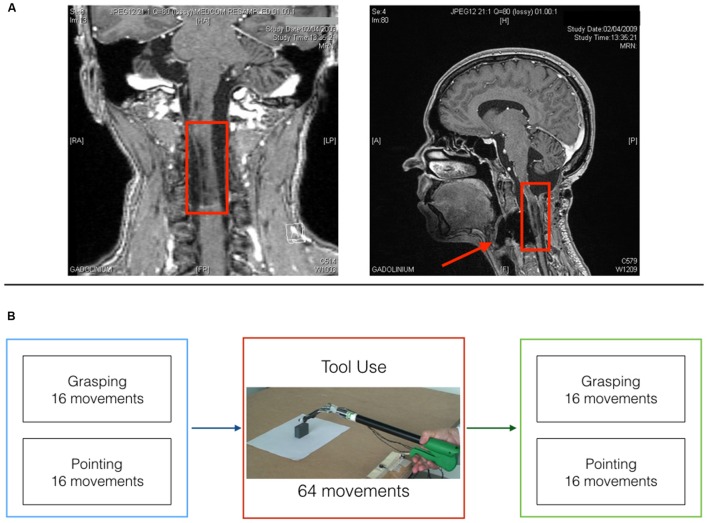
**Patient D.C. post surgery MRI and experimental timeline. (A)** Coronal (left) and midsagittal (right) view of patient D.C.’s post surgery lesion. **(B)** Experimental timeline for the three testing sessions.

Miss D.C., a right-handed 39 years old medical secretary, underwent a surgery in march 2006 for resecting a vascular tumor (hemangioblastoma) at the level of the medulla oblongata on the right side (see post-operative MRI; **Figure 1A**), which left her with no somatosensory sensations from the right upper limb (arthrokinesthesia and pallesthesia were abolished whereas thermalgesic sensitivity was preserved). The patient was hospitalized for a month after surgery in a rehabilitation clinic where she followed a daily physiotherapy program. After being discharged, she continued physiotherapy twice a week for about 1 year time. Before inclusion, clinical examination with the RASP (Winward et al., 2002) found somato-sensory impairment located on the palmar side of the right hand for sharp/dull discrimination (two errors out of eight trials), surface pressure touch (four errors out of eight trials), two-point discrimination (absence of two points discrimination with the 5 mm spacing) and proprioception movement of the index (movement felt one out of six trials without direction stated correctly in none of the six trials). No errors were observed on the temperature discrimination and surface localization sub-tests. The patient had a good motor control of her right hand with a score of 62 out of 66 on the upper-extremity test of the Fugl-Meyer assessment (Fugl-Meyer et al., 1975). She obtained a score of one out of two on the Hand to lumbar spine sub-test, Shoulder flexion to 90°, elbow at 0°, reflex intensity and dysmetria on the coordination sub-test. Somatosensory Evoked Potentials (SEPs) at 1 year from surgery (March 2007) revealed only peripheral response to the medial nerve stimulation at elbow level. Nine months later (January 2008) the same examination revealed a spinal response (N13), but no subcortical or cortical evoked activity. Her ability to perform visually guided movements allowed us to test the role of proprioception in the update of the body representation for action called Body Schema (BS). BS (Head and Holmes, 1911) contains metric body knowledge useful to plan, execute or imagine movements, such as body position in space, size and shape of body parts (Cardinali et al., 2009a; Berlucchi and Aglioti, 2010; de Vignemont, 2010; Medina and Coslett, 2010; Martel et al., 2016). Its existence overlaps with the effector representation which existence is postulated by motor control theories (Miall, 1998; Kawato, 1999; Shadmehr and Krakauer, 2008; Rieger, 2012; Gaveau et al., 2014). Shadmehr and Krakauer (2008, p. 359) indeed suggested that a key goal of motor control systems is “to form a belief about the state of our body and the world (called state estimation).” As we move and perform actions, the state of our body changes, sensory information reaches the brain and the BS is updated to take them into account. Among those sensory signals, proprioception has always been considered the key source of information to maintain an accurate representation of the body. Here, we tested such hypothesis in the case of tool use. Two are the reasons behind the choice of a tool use paradigm: First, tools use is a very important set of skills that pervade almost every aspect of our life. We use tools to eat, for personal hygiene, to work and even in our leisure time to play sports. As a consequence, impairment in such skills or the inability to acquire them has a strong impact on a patient’s life. Providing an insight on the mechanisms supporting tool use in patients that lack proprioception can have important repercussions on the development of rehabilitation programs and new therapeutic approaches. Second, a large amount of literature supports the idea that the extraordinary human ability to use tools relies on the brain ability to incorporate them into the BS (Maravita and Iriki, 2004; Farnè et al., 2007; Arbib et al., 2009; Jacobs et al., 2009; Cardinali et al., 2012). This means that tool use constitutes a perfect paradigm to study BS plasticity and its rules. Indeed, when actions are performed with tools, specific body parts’ morphology and functionality are drastically modified, which requires a quick and efficient update of the BS to maintain action accuracy. We previously showed that when healthy participants reach and grasp objects using a mechanical grabber that functionally elongates their arm, the brain selectively updates the representation of their arm length to take into account the modification induced by the tool (Cardinali et al., 2009b). After using a 40 cm long tool, participants are typically found to act as if their arm was longer than before. In terms of kinematic profile of the movements, post tool-use movements display protracted and reduced peak of wrist acceleration, velocity and deceleration compared to before. Moreover, this pattern has been reported for free-hand pointing movements performed before and after tool-use, but never with the tool. This generalization to untrained movements demonstrates that the origin of the tool-use induced effect is in the lengthening of the arm representation (which would be reflected in changes in any movement performed with that arm) rather than a modification of a specific motor planning for grasping.

While the update is generalized to different movements performed with the specific body-part, it is also specific for the body segment which morphology is modified by the tool. For example, when participants perform grasping actions with pliers that elongate their fingers, only fingers representations are updated (Cardinali et al., 2016). Miller et al. (2014) work also support the limb specificity of BS update. They used a Tactile Distance Judgment task (TDJ) where they stimulated healthy participants in two distinct points, at two different locations (hand/arm and forehead), and asked them to judge where the distance was bigger. The TDJ task was performed before and after using either a classical mechanical grabber, or a hand-shaped tool. After using the arm-shaped grabber, tactile distance perception on the arm was modified, while that on the hand was not.

Several studies indicated that tool incorporation in healthy participants does not require a specific learning process. Indeed, when comparing the first movements with the tool to the last ones, we did not find any kinematic difference, suggesting that BS plasticity supports tool-use by incorporating the tool into the representation of the arm and, by doing so, allowing the brain to control the tool as efficiently as the body.

While some of the rules of tool-use induced BS plasticity have been identified (Cardinali et al., 2016; Martel et al., 2016), the question of which sensory information drives such a plastic mechanism remains unanswered. Here, we tested the role of proprioception by recording D.C.’s kinematics of free-hand grasping and pointing movements before and after a period of tool use in which the patient was asked to use the same grabber as in our previous studies on healthy participants (Cardinali et al., 2009b, 2012). The rationale is that if proprioception is essential to tool incorporation, we should not observe, in patient D.C.’s behavior, the signs of tool incorporation that have been identified in neurotypical subject, at least in the first session and without learning period. Indeed, while previous work on deafferented patients has clearly indicated that motor control can be re-established to a somewhat large extent, especially under visual control (Cole and Paillard, 1995; Ghez and Sainburg, 1995; Lefumat et al., 2016) our hypothesis is that immediate tool incorporation can not happen.

## Materials and Methods

We tested D.C. in three separate and identical sessions between June 2008 and May 2010 after obtaining informed consent to participate in the study, which was conducted in accordance to the Helsinki Declaration and was approved by the local ethics board.

The patient was comfortably seated at a table in a room equipped with a kinematic recording system (Optotrak 2030, Waterloo, ON, Canada; acquisition frequency 200 Hz). Infrared Emitting Diodes were taped on her right thumb (inside corner of the fingernail), index (external corner of the nail) and wrist, as well as on the tip of the tool prongs (see below). A switch on the proximal edge of the table marked the starting position for hand and tool movements. The tool consisted in an ergonomic handle (10 cm-long), a 30 cm-long rigid shaft and an articulated “hand” composed by two curved prongs covered with rubber to assure a stable prehension. The tool was controlled by squeezing the handle with the entire hand and digits: closing the hand in a fist-like posture would bring the tip of the prongs to contact while opening the hand would release the grip.

The patient was asked to perform one block of reach-to-grasp movements and one block of reach-to-point movements before and after four blocks of tool-use (see **Figure 1B**).

In the reach-to-grasp block D.C. was asked to reach out for the target object, grasp it between thumb and index finger (precision grip), lift it, place it back on the table and return to the starting position. In the reach-to-point block, D.C. had to reach out to touch the top of the object with the tip of the index finger and then go back to the starting position. Finally, tool-use consisted in four blocks of reach-to-grasp and lift movements with the grabber. Each block consisted in 16 movements toward two objects of different size (Large or small; eight movements per size).

At the beginning of each trial, the patient was asked to keep both hands on a pinch-grip position (i.e., thumb and index in contact) on two switches. For the tool-use blocks D.C. was asked to keep the tip of the prongs in contact on the same switch. The target object was positioned on the table, in front of the right hand, 36 cm from the starting point. D.C. was instructed to wait for an acoustical go signal to start the movement (depending on the block, i.e., reach-to-grasp, reach-to-point, or tool-use).

Movements were analyzed off line with a custom-made software implemented in Matlab (MATLAB, The MathWorks Inc., Natick, MA, USA, 0.01 mm 3D resolution at 2.25 m distance) and the following kinematic parameters were extracted: wrist Acceleration, Velocity, and Deceleration (both peak amplitude and latency) for the transport component of the movement; Velocity of Fingers Aperture (VFA) and Maximal Grip Aperture (MGA; both amplitude and latency) for the grip component. Parameters were extracted in a semiautomatic fashion: latencies were calculated in milliseconds as the time between movement onset (defined as the first of 20 consecutive frames where the wrist velocity continuously increased) and the time the curve reached its first maximal peak.

## Results

### Session 1

**FIGURE 2:**
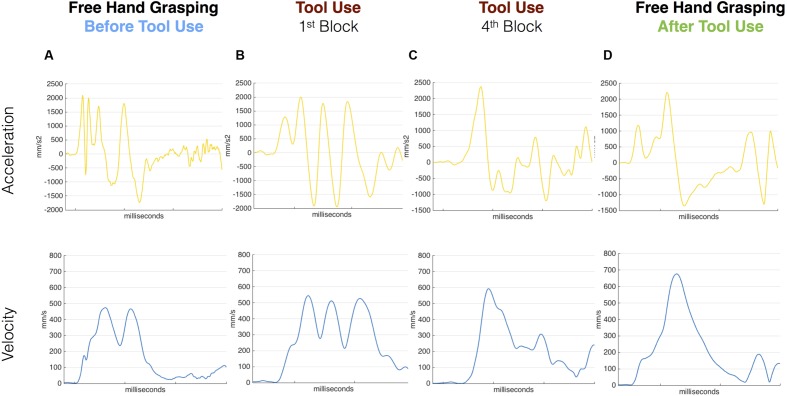
**Acceleration and velocity profile for free-hand and tool grasping movements during Session 1.** Velocity (blue) and Acceleration (yellow) profile of representative movements performed before tool use **(A)**, during the first and last tool blocks **(B,C)** and after **(D)** tool use in Session 1. D.C.’s kinematic profile was characterized by multiple peaks in the pre tool-use phase as well as in the very first block of tool-use. The kinematic profile evolved during the use of the grabber and in the last block D.C. showed a cleaner profile. The same profile was then transferred to the hand in the post-tool use phase where D.C. shows single Velocity and Acceleration main peaks.

In the pre tool-use phase, D.C.’s right hand movements showed an abnormal kinematic profile. While a normal reach-to-grasp movement is characterized by a single peak of acceleration followed by a single peak of velocity and then a peak of deceleration of the wrist, D.C.’s movements showed multiple peaks for all those parameters (**Figure 2A**; See **Supplementary Figure S1** for a comparison between patient D.C.’s velocity profile and a group of controls in the same task). The grip component of the movement seemed to be more affected than the transport component, as 13 out of 16 movements showed double peaks for both VFA and MGA (vs. nine movements with a double peak of velocity; see Supplementary Table S1). Similarly, for reach-to-point movements D.C. showed multiple peaks of acceleration, velocity, and deceleration (in 1, 13, and 15 movements, respectively, out of 16 trials). These results are in line with previous studies showing perturbed motor control in absence of proprioception (Cole and Paillard, 1995; Ghez and Sainburg, 1995; Sarlegna et al., 2006).

The same pattern was visible for the first movements with the tool (**Figure 2B**). When the patient was asked to grasp the target object with the grabber, the kinematic profile of the first movements with the tool was characterized by multiple peaks of acceleration, velocity, and deceleration. Crucially, as the tool-use trials proceeded, this profile progressively evolved (**Figure 2C**). In the last block (16 movements) the patient showed a single peak of velocity per movement and a reduced number of peaks of acceleration and deceleration.

Most interestingly, in the post tool-use phase, D.C.’s kinematic profile for both the free-hand grasping and pointing movements showed a rather normalized pattern, with single peaks of acceleration, velocity, and deceleration in all 16 movements (**Figure 2D**).

Given the qualitative difference in the kinematic patterns of pre- and post-tool-use movements, no quantitative analysis to assess for the presence of tool-incorporation effects was possible at session 1.

### Session 2

**FIGURE 3:**
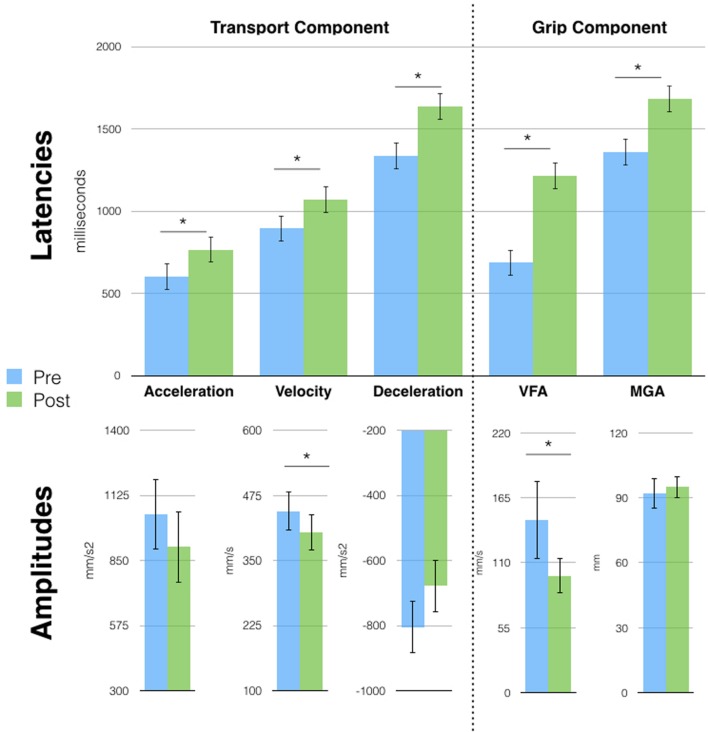
**Free-hand grasping movements were affected after tool-use in session 2.** Patient D.C. showed longer latencies for all parameters and reduced peak amplitude for acceleration and Velocity of Fingers Aperture (VFA). Velocity and Deceleration peaks showed the same tendency but failed to reach significance. Asterisks indicate significant differences, error bars represent SD.

Six months later, the kinematic pattern acquired after Session 1 tool-use phase (single peaks) was still present, suggesting that a short period of tool use had long-lasting effects. We therefore run a series of *t*-tests (Bonferroni corrected) on each parameter of free-hand grasping to compare movements before and after tool-use. Results (**Figure 3**) show that all transport component parameters were affected by tool-use. In particular, we found a significant increase in the latencies of the wrist Acceleration (603 vs. 767 ms, *p* = 0.03), Velocity (895 vs. 1071 ms, *p* = 0.02), and Deceleration (1336 vs. 1636 ms, *p* < 0.01) peaks. Also, the amplitude of the wrist velocity peak was significantly reduced in the post tool-use phase (445 vs. 404 mm/s, *p* = 0.04). Both wrist acceleration and deceleration peaks showed a tendency in the same direction (acceleration: 1046 vs. 908 mm/s2; deceleration -805 vs. -678 mm/s^2^) but did not reach significance (*p* = 0.14 and *p* = 0.08, respectively).

This pattern of results for the transport component is similar to the one observed in previous studies in healthy participants after the use of the same tool (Cardinali et al., 2009b, 2012). At odds with our previous results, D.C.’s grip component was also modified after tool-use. Here, we observed longer latencies for the VFA (687 vs. 1213 ms, *p* < 0.01) and MGA (1360 vs. 1681 ms, *p* < 0.01) and a reduced peak of VFA (66 vs. 99 mm/s, *p* < 0.01). Moreover, tool induced effects did not generalize to the pointing movement, contrary to what was previously reported in healthy participants (**Supplementary Figure S3A**). Indeed, when performing the same pre vs. post tool-use comparison on pointing movements’ parameters, we found no significant difference.

Finally, since tool movements too displayed single peaks, we compared the first with the fourth block to assess for the presence of learning. We found a significant difference for all transport parameters latencies (Acceleration: 220 vs. 440 ms; Velocity: 451 vs. 833 ms; Deceleration: 630 vs. 1062 ms; all *p* < 0.01) and for the amplitude of the acceleration (2859 vs. 1975 mm/s^2^; *p* = 0.02) and deceleration peaks (-1516 vs. -1886 mm/s^2^; *p* < 0.01; see **Supplementary Figure S2**) showing the presence of a learning process.

### Session 3

**FIGURE 4:**
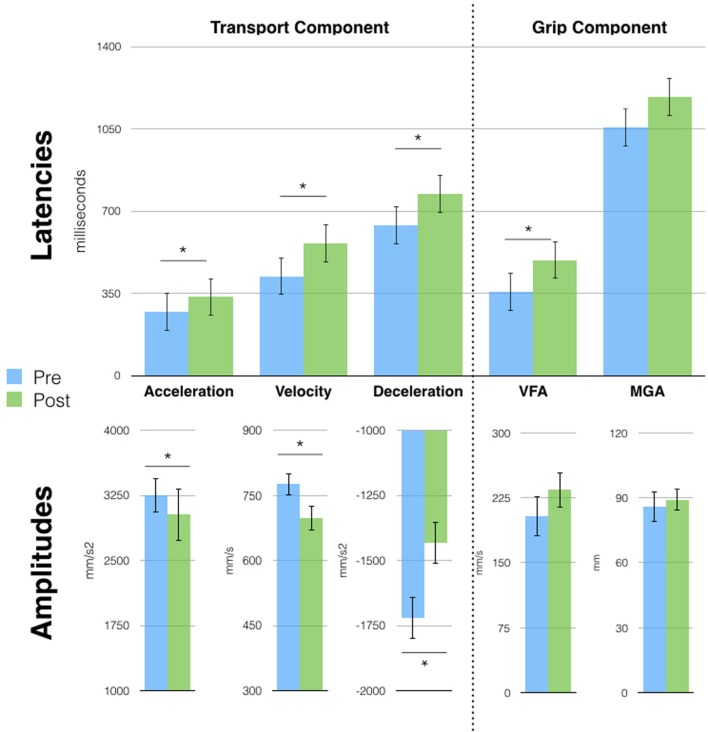
**Free-hand grasping movements were affected after tool-use in session 3.** Patient D.C. showed longer latencies for all parameters [except Maximal Grip Aperture (MGA)] and reduced peak amplitude for the transport component parameters. Asterisks indicate significant differences, error bars represent SD.

Two years and 6 months after session 1, D.C. still exhibited the single-peaked kinematic profile visible right after the very first tool-use phase (session 1). A series of *t*-tests (Bonferroni corrected) on grasping movements parameters, showed increased latencies and a reduction in the amplitude of the peaks of all wrist parameters (all *p* < 0.03) after tool use. The effect on the grip component was, however, reduced, as only the latency of VFA was different after tool use (357 vs. 492 ms, *p* = 0.02: **Figure 4**). Similarly to session 2, no effects were observed on the pointing movements (**Supplementary Figure S3B**).

## Discussion

Here, we tested the long-held hypothesis that proprioception is fundamental for updating the BS (Head and Holmes, 1911). To this aim, we exploited a tool-use paradigm that is known to induce plasticity of the BS, combined with a kinematic approach to test for the presence of such plasticity in D.C., a patient suffering from a deafferented right upper limb. To our knowledge, this is also the first longitudinal study to describe tool-use in a deafferented patient.

Our results suggest that proprioception is fundamental for tool induced BS plasticity: tools cannot be fully and rapidly incorporated when only visual information is available to guide the movement. Indeed, despite showing a different kinematic profile for free-hand grasping after tool-use, D.C.’s motor behavior differed from the kinematic pattern that has been repeatedly reported after use of this mechanical grabber. In healthy participants, the real, or even merely imagined, execution of reach-to-grasp movements with the grabber used in this study brings to changes in several kinematics parameters of free hand actions, with prolonged latencies and reduced peaks (Cardinali et al., 2009b, 2012; Baccarini et al., 2014). Crucially, these changes (1) are body part specific: they affect the transport component in case of tools functionally lengthening the arm, or the grasp component in case of tools affecting the hand morphology/functionality (Miller et al., 2014; Cardinali et al., 2016), (2) they generalize to movements that are not executed with the tool (such as pointing to an object instead of grasping it), and (3) do not require learning (the kinematic profile of tool use does not change during its use in healthy participants). Instead, patient D.C. clearly showed an unspecific pattern: using the long mechanical grabber did not affect the transport component solely, rather altering the grasping component too. Moreover, the changes observed in patient D.C.’s kinematics after tool-use did not generalize to the free-hand pointing movement, again at odds with the pattern previously reported in healthy subjects following use of the same tool. Finally, patient D.C. required a period of learning reflected by the changes in all kinematic parameters as tool-use progressed. The lack of specificity for transport component and of generalization to untrained movements with the presence of motor learning are evidence that what the patient was using to execute the grasping movements was not an updated representation of the arm length, but rather a new motor program developed to control the tool. These differences are more likely to reflect a sensorimotor learning process based on visual feedback. Indeed, previous studies on sensorimotor learning using force fields or visuomotor rotations (Kluzik et al., 2008; Heuer and Hegele, 2015) found that when people adapt to a movement perturbation that is attributed to a change in the arm state the effects are broad modifications that generalize to other movements. This is indeed what, we found in our previous studies on healthy participants (where grasping with a tool affected both subsequent free hand grasping and pointing movements) but not in our patient. In other words, being unable to incorporate the tool and update the representation of the arm length because of the lack of proprioception, D.C. learned a new type of grasping plan which was then recruited after tool-use to perform grasping actions (and those actions only) with her own hand, leading to a new kinematic profile for all components involved in that action (i.e., transport and grip). Indeed, if tool incorporation was not possible without proprioception, we know that motor learning is (Sarlegna et al., 2010; Yousif et al., 2015; Lefumat et al., 2016). The preserved ability to learn motor patterns and transfer them across effectors is our second main result and will be discussed in the next paragraph.

A key and astonishing result is the beneficial effect of a single tool use session on patient D.C.’s free-hand motor behavior. The tool, we asked the participant to use can be seen as a very simplified version of a human arm. It indeed consists in a shaft and two prongs, that is an arm and two fingers, with no wrist articulation. The degrees of freedom of such effector are drastically reduced compared to a real forearm and hand. Moreover, while the functional extremities of the tool allow for a precision grip, the actual control of the tool requires a much less sophisticated power grip on a large handle. In other words, what would require the control of a large number of variables and articulations with her hand, i.e., to reach out and grasp a small object with a thumb-index finger precision grip, can be obtained with a more simplified effector by controlling less variables and implementing a simple motor program. We hypothesize that the simplicity of this effector allowed D.C. to develop a more efficient motor program for reach and grasp actions. The motor program was transferred to the hand in the post tool-use phase, remaining observable for at least the time covered by the two following testing session, about 2 years. This interpretation may have important implications for rehabilitation where the common approach is to restore a rich sensory experience. Our results seem to suggest that patients with sensory deficit may benefit from relatively short periods of practice with simple tools. This could open the path to new affordable and engaging trainings for such patients.

## Author Contributions

LC, CB, JL, AR, and AF designed the experiment. LC and CB conducted the experiment. LC analyzed the data. LC, CB, JL, AR, and AF discussed the results and wrote the paper.

## Conflict of Interest Statement

The authors declare that the research was conducted in the absence of any commercial or financial relationships that could be construed as a potential conflict of interest.

## Supplementary Material

FIGURE S1 **Comparison between patient D.C. velocity profile and a group of healthy controls.** Mean and distribution of velocity peak amplitude (upper panel) and latency (lower panel) for a group of control healthy participants (*N* = 16) performing free-hand grasping movements before (blue curve) and after (green curve) tool use. Bars show the same values for patient D.C.’s across the three testing sessions before (blue) and after (green) tool use.

FIGURE S2 **Tool movements kinematic profile changed from the first (dark red) to the last (light red) block of tool use**. Error bars represent SD.

FIGURE S3 **Free-hand pointing movements were not affected after tool use in sessions 2 and 3**. No significant difference was present for the pointing movements kinematic parameters when comparing between before (blue bars) and after (green bars) tool use. Error bars represent SD.
