# Supplementary figures and images for: Proprioception Is Necessary for Body Schema Plasticity: Evidence from a Deafferented Patient
Source: Front Hum Neurosci. 2016 Jun 16;10:272. doi: 10.3389/fnhum.2016.00272 (PMC4909768; doi:10.3389/fnhum.2016.00272)

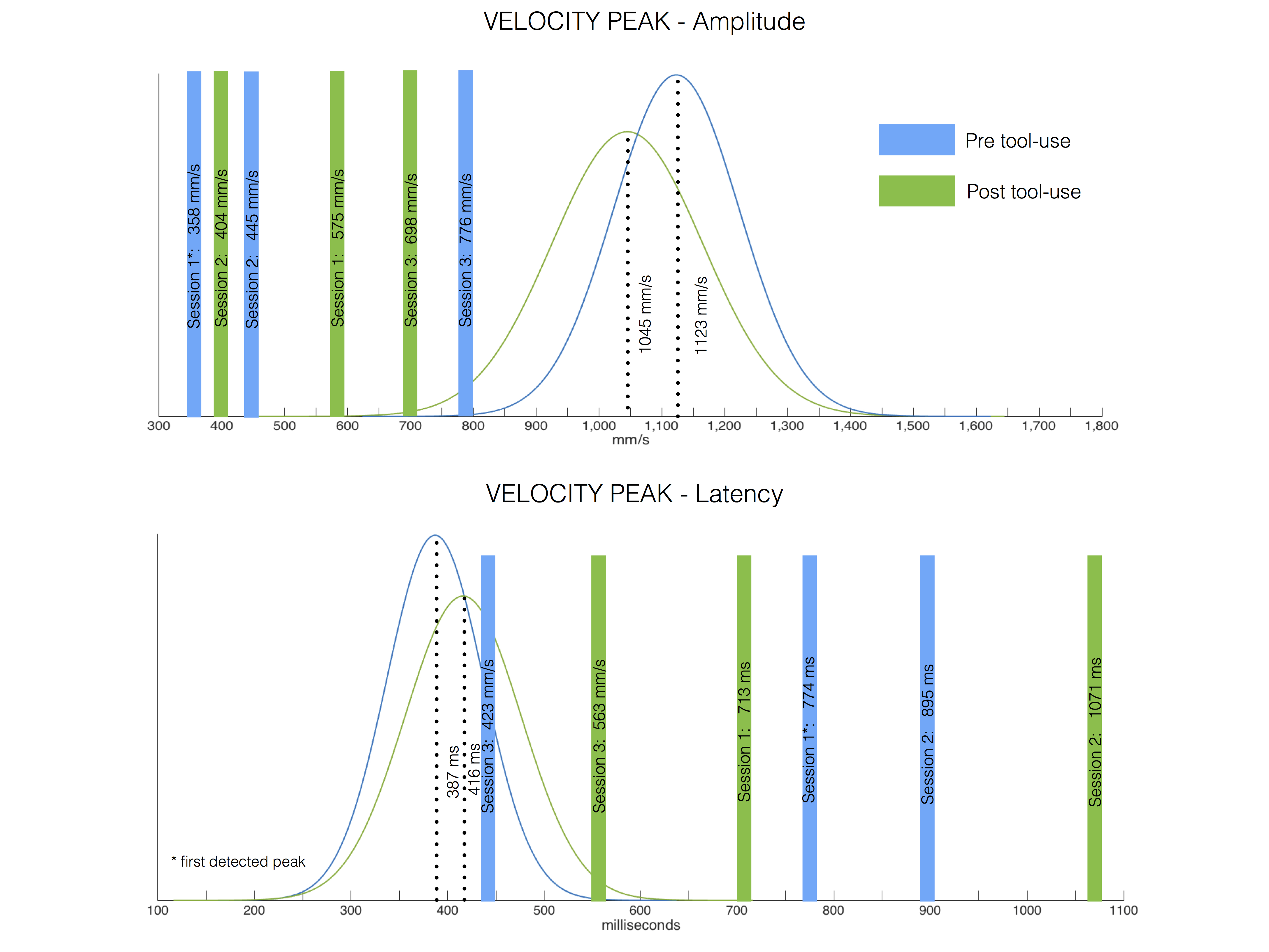

Supplement: FIGURE S1 — Comparison between patient D.C. velocity profile and a group of healthy controls. Mean and distribution of velocity peak amplitude (upper panel) and latency (lower panel) for a group of control healthy participants (N = 16) performing free-hand grasping movements before (blue curve) and after (green curve) tool use. Bars show the same values for patient D.C.’s across the three testing sessions before (blue) and after (green) tool use. [file Image_1.JPEG]

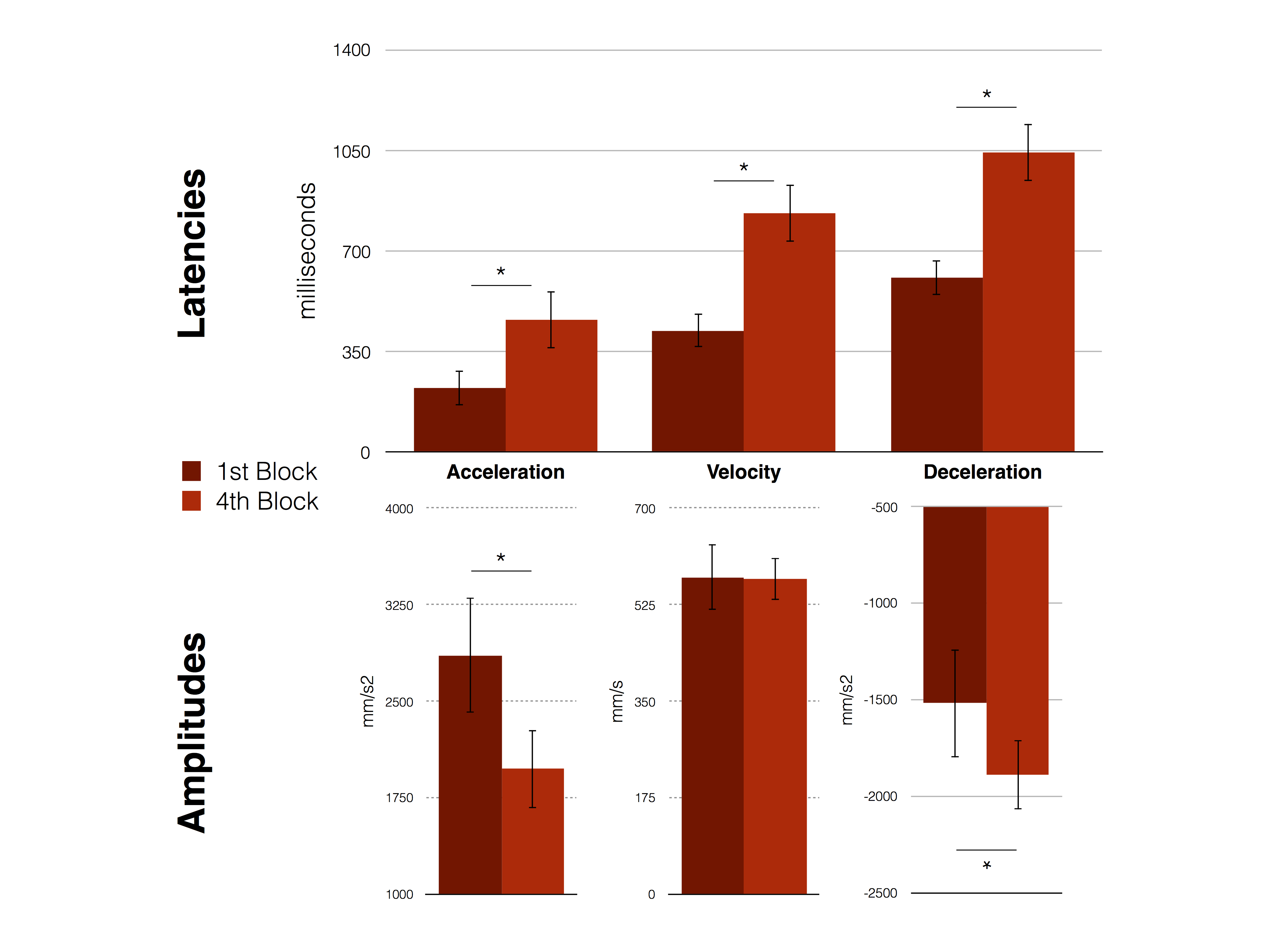

Supplement: FIGURE S2 — Tool movements kinematic profile changed from the first (dark red) to the last (light red) block of tool use. Error bars represent SD. [file Image_2.JPEG]

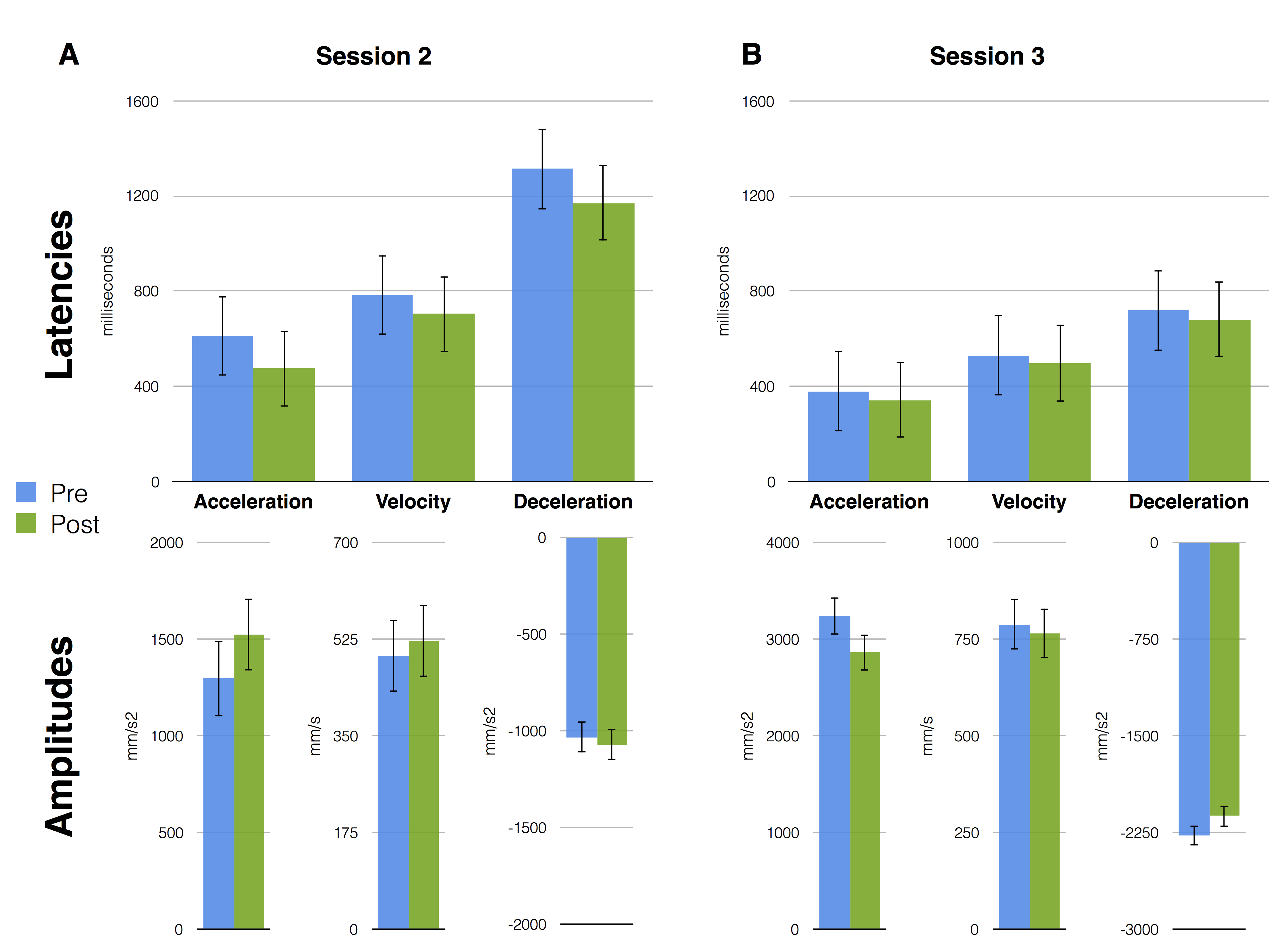

Supplement: FIGURE S3 — Free-hand pointing movements were not affected after tool use in sessions 2 and 3. No significant difference was present for the pointing movements kinematic parameters when comparing between before (blue bars) and after (green bars) tool use. Error bars represent SD. [file Image_3.JPEG]
