# Supplementary material for: Proprioception Is Necessary for Body Schema Plasticity: Evidence from a Deafferented Patient
Source: Front Hum Neurosci. 2016 Jun 16;10:272. doi: 10.3389/fnhum.2016.00272 (PMC4909768; doi:10.3389/fnhum.2016.00272)
Supplement: Supplementary file 4 [file Table_1.PDF]

Supplementary Table 1. Number of trials (out of 16) of Session 1 showing the presence of multiple peaks for each kinematic parameter per movement type.

Movements	Acceleration	Velocity	Deceleration	VFA	MGA
Reach-to-grasp	4	9	7	13	14
Pointing	1	13	15	-	-
